# Identification and classification of antiviral defence systems in bacteria and archaea with PADLOC reveals new system types

**DOI:** 10.1093/nar/gkab883

**Published:** 2021-10-04

**Authors:** Leighton J Payne, Thomas C Todeschini, Yi Wu, Benjamin J Perry, Clive W Ronson, Peter C Fineran, Franklin L Nobrega, Simon A Jackson

**Affiliations:** Department of Microbiology and Immunology, University of Otago, Dunedin, New Zealand; School of Biological Sciences, Faculty of Environmental and Life Sciences, University of Southampton, Southampton, UK; School of Biological Sciences, Faculty of Environmental and Life Sciences, University of Southampton, Southampton, UK; Department of Microbiology and Immunology, University of Otago, Dunedin, New Zealand; Department of Microbiology and Immunology, University of Otago, Dunedin, New Zealand; Genetics Otago, University of Otago, Dunedin, New Zealand; Department of Microbiology and Immunology, University of Otago, Dunedin, New Zealand; Genetics Otago, University of Otago, Dunedin, New Zealand; Bioprotection Aotearoa, University of Otago, Dunedin, New Zealand; Maurice Wilkins Centre for Molecular Biodiscovery, University of Otago, Dunedin, New Zealand; School of Biological Sciences, Faculty of Environmental and Life Sciences, University of Southampton, Southampton, UK; Department of Microbiology and Immunology, University of Otago, Dunedin, New Zealand; Genetics Otago, University of Otago, Dunedin, New Zealand; Bioprotection Aotearoa, University of Otago, Dunedin, New Zealand; Maurice Wilkins Centre for Molecular Biodiscovery, University of Otago, Dunedin, New Zealand

## Abstract

To provide protection against viral infection and limit the uptake of mobile genetic elements, bacteria and archaea have evolved many diverse defence systems. The discovery and application of CRISPR-Cas adaptive immune systems has spurred recent interest in the identification and classification of new types of defence systems. Many new defence systems have recently been reported but there is a lack of accessible tools available to identify homologs of these systems in different genomes. Here, we report the Prokaryotic Antiviral Defence LOCator (PADLOC), a flexible and scalable open-source tool for defence system identification. With PADLOC, defence system genes are identified using HMM-based homologue searches, followed by validation of system completeness using gene presence/absence and synteny criteria specified by customisable system classifications. We show that PADLOC identifies defence systems with high accuracy and sensitivity. Our modular approach to organising the HMMs and system classifications allows additional defence systems to be easily integrated into the PADLOC database. To demonstrate application of PADLOC to biological questions, we used PADLOC to identify six new subtypes of known defence systems and a putative novel defence system comprised of a helicase, methylase and ATPase. PADLOC is available as a standalone package (https://github.com/padlocbio/padloc) and as a webserver (https://padloc.otago.ac.nz).

## INTRODUCTION

Bacteria and archaea possess a variety of defence systems to protect against diverse types of phages and mobile genetic elements (MGEs) (1,2) and to limit phages and MGEs from evading defence (3,4). The discovery and characterisation of novel defence systems has increased our understanding of the interactions between phages or MGEs and their hosts, and has led to the discovery of unique enzyme functionality that has been repurposed for new molecular tools, such as Cas9 for genome editing (5–7). Within genomes, defence systems are often concentrated in distinct genomic loci termed ‘defence islands’ (8,9). Many new types of defence systems have recently been discovered by studying the genomic ‘dark matter’ of defence islands using a guilt-by-association approach—uncharacterised genes that commonly reside next to genes of known phage defence systems often encode novel defence systems (10–14). As more genomic data are deposited into sequence databases, there are also renewed efforts to comprehensively identify and characterise known defence systems (15–19).

Several software tools have been developed to identify defence systems in prokaryotic genomes (20–26). However, these tools are often tailored to identify specific types of defence systems, such as CRISPR-Cas. Several precomputed databases are available for other defence systems including toxin-antitoxin and restriction-modification systems (27–31). However, these databases are limited to publicly available data and most lack the capability of searching user-supplied genomes on demand. As new types of defence systems are discovered and existing defence system classifications are revised, software tools will need to adapt to use this new information. To address the lack in capability of current tools to identify many types of phage defence systems, we have developed a scalable, open-source Prokaryotic Antiviral Defence LOCator (PADLOC). PADLOC is available as a standalone package (https://github.com/padlocbio/padloc) and as a webserver (https://padloc.otago.ac.nz). Both resources allow analysis of user-supplied genomes, and the webserver includes precomputed PADLOC results from the RefSeq Bacteria and Archaea genome database (32).

Since most phage defence systems function through the coordinated action of multiple proteins that are encoded together in a single genomic locus, PADLOC uses a modified implementation of an approach previously developed to identify multi-gene macromolecular systems (20). Briefly, genes encoding defence system homologues are identified using profile Hidden Markov Models (HMMs), followed by validation of defence system completeness using gene presence/absence requirements specified in defence system classification files. For the initial release of PADLOC, we focused on two large groups of recently discovered phage defence systems, those identified in Doron *et al.* (13) (Druantia, Gabija, Hachiman, Kiwa, Lamassu, Septu, Shedu, Thoeris, Wadjet and Zorya, hereafter the ‘Doron systems’) and the cyclic-oligonucleotide-based anti-phage signalling system (CBASS) classifications described in Millman *et al.* (18). The Doron systems include the Wadjet systems that provide plasmid defence and are equivalent to the efficient plasmid transformation (*ept*) systems discovered in *Mycobacterium smegmatis* (33). We demonstrate that PADLOC can be used to identify phage and plasmid defence systems in prokaryotic genomes with high accuracy and specificity. In addition, we have used PADLOC to discover several new variant types of Doron systems, providing a foundation for further functional research. For future scalability, we used a modular approach for the organisation of HMMs and system classifications, which allows new defence systems to easily be added to the PADLOC defence systems database. As such, PADLOC provides a framework for continued community development into an all-in-one tool to identify the rapidly expanding set of known defence systems.

## MATERIALS AND METHODS

### PADLOC implementation

**Figure 1.**
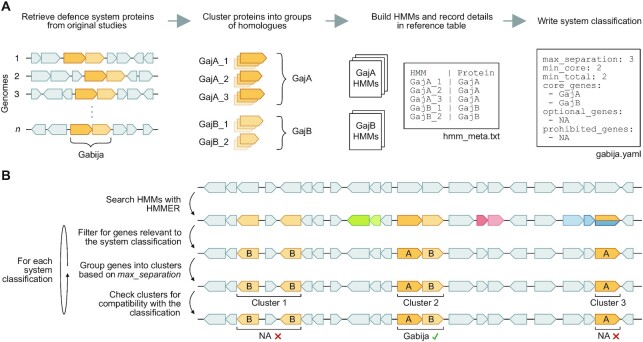
Workflow of data preparation and PADLOC functioning. (**A**) Preparation of data for PADLOC. For each type of defence system protein, sequences were retrieved and clustered into homologue groups. An HMM was built from each group of proteins, and the names of the HMMs (e.g. GajA_1) and their corresponding protein families (e.g. GajA) were recorded in a reference table (hmm_meta.txt), which allows a single family of defence system proteins to be represented by multiple HMMs. A simple classification file ([system].yaml) was written to represent each defence system, describing the typical genetic architecture of the system. (**B**) Automated functional workflow of PADLOC. HMMER is used to identify genes encoding defence protein homologues in the input genome. Each system classification is then analysed individually, filtering the HMM hits for genes relevant to the current type of system being searched. HMM hits are grouped into gene clusters based on the synteny requirements specified in the system classification. Each cluster is then checked against the system classification to determine whether the system requirements are fulfilled. Yellow genes represent Gabija; green, red or blue genes represent genes from other defence systems; genes with two colours (i.e. yellow/blue) represent genes matched by HMMs from two different defence systems.

PADLOC uses a protein FASTA and corresponding Generic Feature Format (GFF3) file as input, which are commonly generated by genome annotation pipelines including the NCBI Prokaryotic Genome Annotation Pipeline (34), IMG Annotation Pipeline (35) and Prokka (36). Alternatively, a nucleotide FASTA file can be supplied as input, in which case Prodigal (37) is used to predict open reading frames and produce a protein FASTA and GFF3 file. Defence system proteins are identified using profile Hidden Markov Models with HMMER (38). Defence system classifications are described in YAML (a simple data serialisation language) formatted files (see Figure 1A, Supplementary Figure S1A and the PADLOC database GitHub repository https://github.com/padlocbio/padloc-db, for example system classification structure). All HMMs and classification files are available from the PADLOC database GitHub repository. In the YAML system classifications, proteins are designated as *core, optional or prohibited*. *Core* proteins are those expected to be present for a functional system. Proteins classed as *optional* are not strictly required for system identification. Specifying proteins as *prohibited* is useful when distinguishing between similar types of systems that may share *core* components but differ by a few key proteins. For each classification, a minimum number of *core* genes (*minimum_core*) and total *core/optional* genes (*minimum_total*) must be satisfied. When the requirements for a system are met, the location and details of the relevant corresponding genes are recorded as output. A simplified GFF file is also generated, allowing for annotation of the defence systems in genome viewing software. The typical run time of PADLOC for a genome encoding ∼4,500 proteins is less than one minute.

### Building HMMs, system classifications and benchmarking

To build profile HMMs for the Doron and CBASS system proteins, we first retrieved the relevant protein sequences from defence system loci listed by Doron *et al.* (13) and Millman *et al.* (18). Redundant identical sequences were removed using SeqKit v0.13.2 (39). The sequences of each protein were then clustered at 30% minimum sequence identity and 80% alignment coverage with MMseqs2 v12.113e3 (40). If a cluster contained >100 sequences, redundancy was reduced at a threshold of 90% sequence identity and 90% pairwise alignment coverage with CDHIT v4.8.1 (41) using the accurate/slow clustering mode. Clusters with less than 200 sequences were aligned with MUSCLE v3.8.1551 (42) with anchor optimisation disabled and clusters with >200 sequences were aligned with MAFFT v7.471 (43) using one guide tree. An HMM was built for each cluster with at least five sequences using HMMER v3.3 with default parameters. PADLOC system classifications were written (in YAML format) to represent reported Doron and CBASS types/subtypes (13,18). In most cases the HMM scoring cut-offs for *E*-value and alignment coverage were set at 1 × 10^–5^ and 30%, respectively. For the single-gene Shedu system, *E*-value and alignment cut-offs were set at 1 × 10^–25^ and 50%, respectively. To benchmark the performance of PADLOC, we searched for Doron systems in the genomes listed in the original study and compared the accuracy of defence system recall to what was previously reported (13). To comprehensively identify Doron and CBASS defence systems in publicly available genomes, we used PADLOC to search all RefSeq v201 Archaea and Bacteria genomes (*n* = 192,371, July 2020) (32).

### Identification of new defence system variants

To discover variants of Doron defence systems, we used a subset of RefSeq genomes (*n* = 41,470) with reduced redundancy, comprised of up to five genomes for each bacterial and archaeal species defined by either the NCBI or GTDB taxonomy (44). We first attempted to identify variants by searching for orphan (single) Doron system genes. This approach proved unproductive because the Doron systems often comprise proteins with nuclease, helicase and/or ATPase domains, which are abundant in prokaryotic genomes and implicated in a variety of functions. As a result, our search revealed a large number of hits to proteins unlikely to be related to defence. To reduce the number of false positives, we instead used PADLOC to search for gene clusters that encoded at least two canonical proteins from the Doron systems. Proteins that were encoded within three open reading frames (ORFs) either side of the identified gene clusters were then pooled to give a set of putative defence-associated proteins. Proteins that were already part of a Doron system were removed. We then clustered the putative defence-associated proteins into groups of homologues using MMseqs2 and built HMMs with HMMER (as above). The resulting HMMs were then grouped into larger protein families based on an all-against-all HMM-HMM comparison using HH-suite v3.1.0 (45) with cut-offs of 95% probability and 75% pairwise alignment coverage. We then calculated the frequency of association between each defence-associated protein family and canonical Doron system protein. For two proteins ‘*A*’ and ‘*B*’, the frequency of association was calculated as frequency = (loci encoding A and B)/(loci encoding A). To reduce bias from overrepresented loci (due to many similar genomes from closely related strains), only one representative locus was counted per distinct gene cluster (refer to Supplementary Figure S2A for more details). Defence-associated protein families were filtered for those above a threshold of >50 associations (loci) with at least two different canonical Doron system proteins with a frequency of association >0.5, and at least one with a frequency greater than 0.7; these cut-offs were determined empirically by inspection of the resulting network graphs. The remaining associations were then manually inspected for loci displaying features characteristic of defence systems, including conserved operon-like architecture, presence in diverse genetic contexts and indications of horizontal gene transfer (presence in multiple species). Candidate defence system variants that had indiscernible locus architectures or lacked context diversity were excluded from further analysis (Supplementary Figure S2B). To assign putative function to each Doron system–associated protein, pairwise comparison of their HMMs against PFAM v33 (46) and COG v1 (47) was carried out using HHpred (48).

### Prevalence and phylogeny of defence systems

We analysed the prevalence of all CBASS, canonical Doron systems and candidate new system types by searching all RefSeq v201 Archaea and Bacteria genomes with PADLOC. To investigate the phylogeny of the new Doron system subtypes, we built trees of the shared core components of these systems, i.e. the proteins present in both the canonical and new subtypes. For each type of core protein, the sequences of all the proteins identified with PADLOC were clustered with MMseqs2 and filtered for the top *n* clusters containing ∼90% of the total sequences. Five random sequences were sampled from each of these clusters as representatives of each core protein. These sequences were aligned using MUSCLE and phylogenetic trees were inferred with IQ-TREE v2.0.3 (49) using the best-fit model selected by ModelFinder (50) and ultrafast bootstrap with 1000 replicates (51).

### Phenotypic analysis of defence systems

We assessed the activity of three new Doron system subtypes (Zorya type III, Hachiman type II, and Lamassu type II) *in vivo*. The systems were amplified from the genomic DNA of *Stenotrophomonas nitritireducens* DSM 12575 (NZ_LDJG01000021.1, Zorya type III), *Sphingopyxis witflariensis* DSM 14551 (NZ_NISJ01000011.1, Hachiman type II) and *Janthinobacterium agaricidamnosum* DSM 9628 (NZ_HG322949.1, Lamassu type II) using primers (Integrated DNA Technologies) listed in Supplementary Table S1 with Q5 DNA polymerase (New England Biolabs). The systems were cloned by restriction digestion with enzymes KpnI and BamHI (Zorya type III) or SbfI-HF and NotI-HF (Hachiman type II and Lamassu type II) (New England Biolabs) into a derivative of pACYCDuet-1 (pUOS001 or pUOS0014, Supplementary Table S2) amplified with primers FN0031, FN0032, FN0126, FN0127 (Supplementary Table S1) to introduce the restriction sites. After confirmation by Sanger sequencing (Eurofins Genomics), plasmids pZorya (pUOS004), pHachiman (pUOS002) and pLamassu (pUOS003) (Supplementary Table S2) were transformed into *Escherichia coli* BL21-AI (New England Biolabs), which naturally lacks the three defence systems, for subsequent phage challenge assays.

### Phage propagation and plaque assays

*Escherichia coli* phages T1, T3, T4, T7 and Lambda-vir were obtained from the Fagenbank (Delft, Netherlands). *Salmonella* phage PVP-SE1, previously shown to infect *E. coli* strain BL21-AI (52), was kindly provided by the Azeredo Lab (University of Minho, Braga, Portugal). Phages were propagated on *E. coli* BL21-AI using the plate lysate method (53). The lysate titre was determined using the small drop plaque assay (54). For plaque assays, overnight cultures of BL21-AI containing pZorya, pHachiman or pLamassu were diluted in Lysogeny Broth (LB) supplemented with 25 μg ml^–1^ of chloramphenicol, 1 mM of IPTG and 0.2% (w/v) of L-arabinose and grown to early log phase (OD_600_ of ∼0.3) at 37°C with shaking at 200 rpm. Bacteria were mixed with LB top agar (0.6% (w/v) agar) supplemented with 1 mM IPTG and 0.2% l-arabinose, and with 10-fold serial dilutions of the phages. The mixture was poured on top of LB agar plates (1.5% (w/v) agar) and incubated at 37°C overnight. The efficiency of plaquing (EOP) was determined by comparing plaque formation in bacteria containing the defence systems with that in control bacteria with the empty vector.

### Infection dynamics in liquid medium

Overnight cultures of bacteria containing a new Doron system subtype or control empty vector were diluted in LB supplemented with 25 μg ml^–1^ chloramphenicol, 1 mM IPTG, and 0.2% (w/v) l-arabinose. Cells were grown to early log phase (OD_600_ of ∼0.3), then diluted to a final OD of ∼0.1 and distributed into the wells of a 96-well plate. Phage PVP-SE1 was added to the wells at a multiplicity of infection (MOI) of 0.1 and 0.01. Infections were performed in biological triplicates, and a control without phage (MOI = 0) was used to determine normal bacterial growth. The OD_600_ was monitored every 20 min for 15 h at 37°C using a CLARIOstar Plus plate reader.

## RESULTS

### Defence system identification using profile HMMs and system classifications

To identify genes encoding defence system proteins, we use profile HMM-based homologue detection. HMMs are linked with their corresponding defence system proteins using a reference table (hmm_meta.txt), which includes the minimum requirements for E-value, and target/HMM alignment thresholds for each HMM (Supplementary Figure S1). Using this approach, each protein family can be represented by multiple HMMs. Each type of defence system is defined using a classification file ([system].yaml) that describes the typical genetic architecture of the system, including which genes are required and the maximum allowance for intervening non-system genes. The typical workflow for adding a new type of defence system to PADLOC involves retrieving the appropriate protein sequences, clustering based on sequence similarity, aligning and building profile HMMs, assigning the HMMs to their respective proteins, and writing a [system].yaml classification file to represent the system (Figure 1A). These data can then be used with PADLOC to search for the system. After a genome is searched for defence system genes, each system classification is analysed individually. First, the putative defence genes are filtered for those relevant to the current system classification being analysed (i.e. they have been labelled as core, optional or prohibited) (Figure 1B). Since different defence systems can comprise similar components, this pre-filtering prevents similar HMMs that belong to different system types from affecting defence system detection. The relevant genes are then grouped into gene clusters based on a maximum allowable number of unrelated genes separating them, defined by the *maximum_separation* parameter in the classification file (Figure 1B). Lastly, each gene cluster is checked against the system classification, to determine whether the system requirements are fulfilled. Gene clusters meeting the specifications are reported as encoding the corresponding defence system.

We first validated our PADLOC approach using the Doron defence systems (13). Of the systems reported in the GenBank 2016 dataset by Doron *et al.* (13), we reserved half the data as a testing set and used the remaining half to construct HMMs, then wrote PADLOC-formatted system classifications to define each system subtype. Running PADLOC over both the test and training sets demonstrated a high recall sensitivity (typically >97% recall) (Supplementary Figure S3A). We then expanded the PADLOC dataset to include all data from Doron *et al.* (13) and analysed the detection of systems by PADLOC compared to the reported systems, which revealed a high recall and an increased sensitivity to identify several additional examples of most defence systems (Supplementary Figure S3B). For the single-gene Shedu system, we had to trade-off detection sensitivity for specificity by enforcing higher HMM scoring cut-offs, which resulted in detection of approximately 89% of the Shedu systems listed by Doron *et al.* (13) The sensitivity/specificity trade-off is a limitation of using PADLOC (or indeed any approach relying on profile HMMs to detect single proteins) to identify single gene defence systems and users should note that the accuracy of PADLOC for single-gene systems will be less than for multi-gene systems. Overall, these results demonstrate the quality of our protein models and system classifications and validate the ability of PADLOC to identify defence systems in prokaryotic genomes.

### Identification of new defence system variants

**Figure 2.**
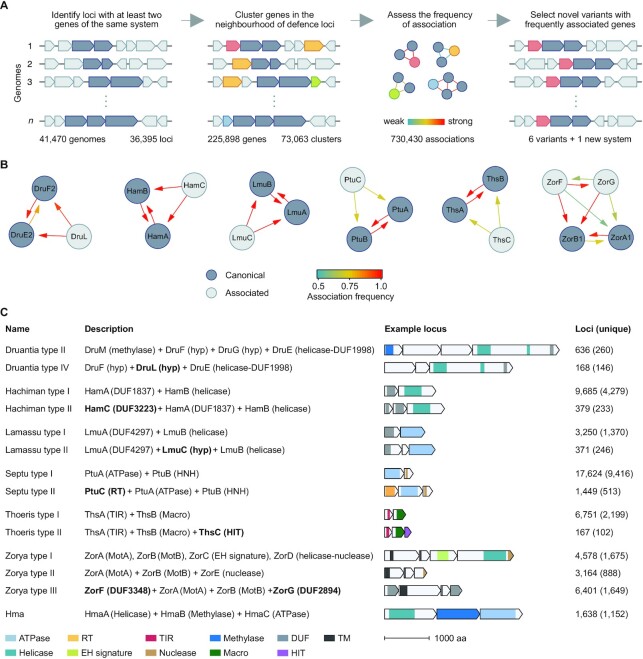
Analysis of proteins associated with Doron systems reveals new system types. (**A**) Workflow of defence system variant identification. PADLOC was used to identify loci encoding Doron system proteins. The proteins encoded by up to three genes either side of each Doron system locus were clustered into families. The frequency of association of each protein family to each Doron system protein was analysed. Loci with frequent associations, conserved locus architecture and found in diverse genetic contexts were considered as new subtypes. (**B**) Network of defence gene associations after filtering for abundance greater than 50 distinct loci, association frequency greater than 0.5, conservation of genetic architecture and context variability. Arrow direction represents association frequency of protein ‘*A*’ (start of arrow) with protein ‘*B*’ (end of arrow). (**C**) Descriptions and schematic diagrams of the new Doron system types and their most similar canonical Doron system types. Domains: RT, reverse transcriptase; DUF, domain of unknown function; TIR, Toll-interleukin receptor; TM, Transmembrane; Hyp, hypothetical protein. Proposed system names: Hma; helicase, methylase and ATPase. Descriptions of proteins that differ between canonical Doron systems and the new types are shown in bold. Refer to Supplementary Table S3 for details of loci examples.

Several types of defence systems have accessory proteins that can regulate, diversify or enhance the antiviral response (18,55,56). For instance, a recent analysis of CBASS systems revealed several distinct types/subtypes that share conserved components, with each type encoding several different ancillary proteins (18). Likewise, we hypothesised that additional subtypes of the Doron systems existed which had not been previously classified. To test this, we systematically identified genes that were frequently associated with each of the Doron systems (Figure 2A). First, we identified 36,395 loci comprised of co-localised genes encoding two or more proteins of the same Doron system, in a set of 41,470 representative genomes. We then clustered the 225,898 proteins encoded by the genes surrounding these defence gene clusters into 73,063 groups of homologues. We then calculated the frequency of association of each group of homologues to each Doron system gene, thereby revealing any protein families with frequent association to each type of Doron system (Figure 2B and Supplementary Figure S4). Loci containing these frequent associations were examined for features characteristic of defence systems (i.e. conserved operon-like genetic architecture, diverse genetic contexts, distribution across distantly related organisms). To further demonstrate that the additional genes of the new subtypes were associated with their respective systems, we searched for cases where the additional genes were present without their respective canonical genes (i.e. orphan occurrences of the subtype-specific genes). In general, the Doron-associated genes were identified more often as belonging to the new system subtypes than they were identified as orphan genes, and in all cases the observed associations were significant (*P* < 0.001, determined using one-sample proportion tests), suggesting functional association (Supplementary Figure S5). Additionally, genes identified as orphans generally were matched by their respective HMMs with a higher *E*-value (i.e. were weaker hits), indicating that the orphan genes were more divergent (Supplementary Figure S5). Altogether, the associations observed between the genes of these systems were robust and, using this method, we identified six putative new Doron system subtypes and an additional putative novel defence system, which we named Hma (Figure 2C).

To comprehensively identify the new Doron system subtypes and the Hma system, we wrote system classifications for PADLOC and searched for them in all RefSeq v201 Archaea and Bacteria genomes (Figure 2C). Altogether, we identified 168 instances of a Druantia-like system that, similar to Druantia type II, encodes DruE and DruF. However, the Druantia-like system lacks the type II requisite DruM and DruG proteins, instead encoding a hypothetical protein with no domain annotations, hereafter named DruL. As such, we have classified this system as a new type of Druantia, type IV. About 3.8% of Hachiman systems (379 systems) were associated with a gene encoding a DUF3223 protein (HamC) either upstream or downstream of *hamAB*. We refer to the new Hachiman systems as type II, and the original Hachiman systems as type I. Similarly, an additional hypothetical protein (LmuC) was identified in about 10.2% of the Lamassu systems detected (371 systems) (Lamassu type II). About 7.6% of Septu systems identified (1,449 systems) were preceded by a gene encoding a reverse-transcriptase (PtuC). This same gene cluster of *ptuC* (reverse-transcriptase), *ptuA* (ATPase) and *ptuB* (HNH endonuclease) was identified previously using several different approaches (14,57,58), and characterised as a retron phage defence system. Our mutual discovery of this association adds support to our method of system variant/subtype detection. Due to the similarity of this system with canonical Septu, we classified it as Septu type II. In 2.4% of Thoeris systems (167 systems), there was an additional gene encoding a histidine triad (HIT) domain protein (ThsC) (Thoeris type II). In addition to the 7,742 canonical Zorya systems found (types I and II), we identified 6,401 pairs of *zorBC* genes flanked by genes encoding a DUF3348 domain protein (ZorF) and a DUF2894 domain protein (ZorG), which we have named Zorya type III. While analysing genes associated with Doron systems, we also identified 1,638 instances of a three-gene operon that occurred frequently with Septu type I systems but was also often found elsewhere (not near Septu) in the genomes analysed. We designated this as a new candidate defence system named Hma, as it encodes three proteins with predicted helicase (HmaA), m5c methyltransferase (HmaB) and ATPase (HmaC) domains.

### Doron system variants provide protection against phage infection

**Figure 3.**
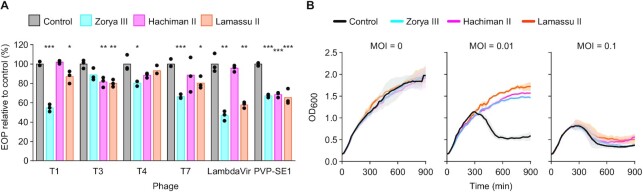
The new Doron system subtypes provide protection against phage infection. (**A**) The efficiency of plaquing (EOP) for *E. coli* BL21-AI possessing representative Zorya type III, Hachiman type II or Lamassu type II systems from *Stenotrophomonas nitritireducens* DSM 12575, *Sphingopyxis witflariensis* DSM 14551 and *Janthinobacterium agaricidamnosum* DSM, respectively, relative to the empty vector control. Graphs show the mean of three biological replicates with individual data points overlaid. Two-sided *t*-test; * *P* < 0.05, ** *P* < 0 .01, *** *P* < 0.001. (**B**) Liquid culture infection time courses for BL21-AI strains possessing the Doron defence system variants, infected with phage PVP-SE1. Growth curves represent the mean of three biological replicates, the shaded area corresponds to the standard error of the mean.

To determine whether the new Doron system subtypes defend against phages, we cloned representative Zorya type III, Hachiman type II and Lamassu type II systems onto plasmids for inducible expression in *E. coli*. We then challenged the bacteria with several types of phages (Siphoviridae: T1, LambdaVir; Myoviridae: T4, PVP-SE1; Podoviridae: T7) (Figure 3). In all cases, we observed significant reductions in the efficiency of plaquing for at least one phage tested for each system (Figure 3A). We also tested a phage (PVP-SE1) with liquid culture experiments at a range of different multiplicities of infection (MOI), and found that each of the systems provided protection at MOI <0.1, demonstrated by an absence of culture collapse upon infection (Figure 3B). These results confirm that Zorya type III, Hachiman type II and Lamassu type II encode functional defence systems.

### The Doron system variants are found in multiple lineages

**Figure 4.**
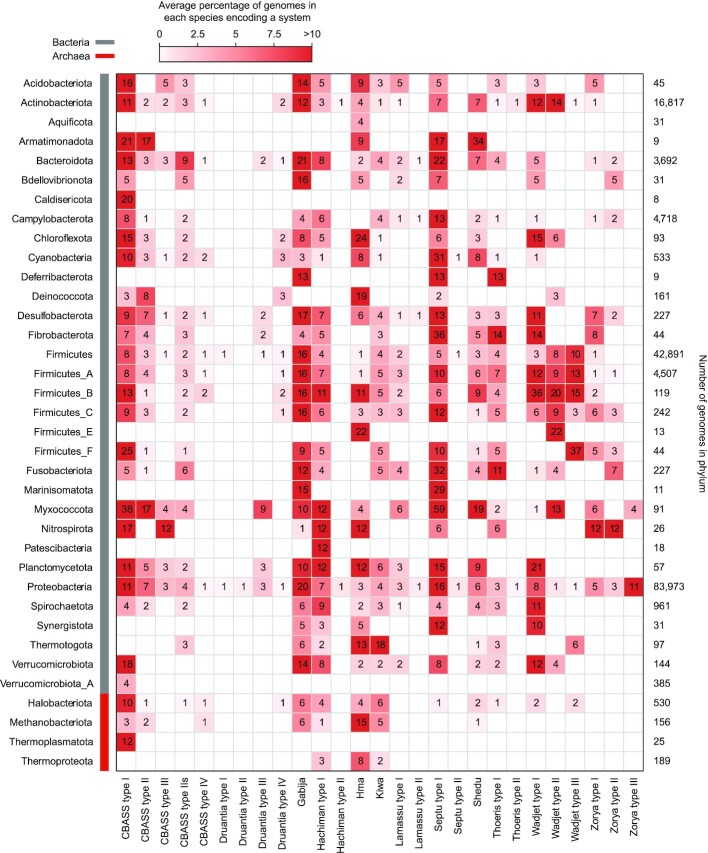
Abundance of defence systems identified with PADLOC in bacteria and archaea. All genomes from RefSeq v201 Archaea and Bacteria were searched with PADLOC. The values in the boxes represent, for each phylum, the average percentage of genomes in each species encoding a system, grouped using GTDB taxonomy (44); system prevalence is weighted in this way to limit biases in phyla that contain many closely related genomes of the same species. The colouring in each box provides a visual representation of these values. Shown are phyla with more than five genomes and at least one type of system. A species-level comparison is provided in Supplementary Figure S6 and the full data are provided in Supplementary Table S5.

Defence systems are frequently transferred between prokaryotes via horizontal gene transfer (2,59,60). As a result, some defence systems are phylogenetically widespread, while others may be confined to specific taxa but show patchy distribution between closely related species (59). To further investigate the new Doron defence system subtypes, we analysed their prevalence and phylogenetic distribution in all RefSeq v201 Archaea and Bacteria. For comparison, we included the canonical Doron systems and also built HMMs and wrote PADLOC system definitions for the recently discovered CBASS systems described in Millman *et al.* (18). We identified each of our new system types in multiple phyla, as is expected for phage defence systems and observed for the canonical Doron and CBASS systems (Figure 4). Druantia type IV was the most widespread of the new Doron system subtypes, present in 11 phyla compared to the related canonical Druantia type II system, which was identified only in Proteobacteria. The putative Hma system was very widespread, present in 26 phyla, surpassed only by CBASS type I, Gabija and Septu type I. At the genus level, the defence systems also exhibited patchy distribution (Supplementary Figure S6), indicative of horizontal transfer, congruent with the function of these systems as phage defences. To determine whether the new Doron system subtypes were divergent from those of the archetypal systems, we analysed the sequence similarity of their core components (i.e. the proteins present in both the new and canonical types) (Supplementary Figure S7, Table S4). In most cases, the core components of the new subtypes were divergent from those of the canonical systems, being present in the same or closely related clans. This sequence divergence could correlate with the acquisition of the additional gene(s) followed by subsequent functional specialisation, although this remains to be determined. Overall, each new defence system subtype exhibited typical defence system characteristics including conserved operon-like genetic architecture, presence in diverse genetic contexts and distribution across distantly related organisms.

## DISCUSSION

Many diverse defence systems have evolved in bacteria and archaea to defend against phages and other MGEs (1). Recently, there has been a surge in the discovery of new types of phage defence systems. However, the systematic identification and annotation of defence systems remains a challenge for biologists interested in searching the genome of their organism of interest. To address the lack in capability of current tools to identify newly discovered types of phage defence systems, we developed PADLOC. When benchmarked against the genomes searched by Doron *et al.* (13), PADLOC detected on average 97% of the multi-gene systems listed in the original study, with some additional systems detected. This demonstrates that PADLOC can identify multi-gene defence systems with high accuracy and specificity. One limitation of PADLOC is that, due to the constraint of genetic synteny, defence systems that are split by breaks in contigs will not be detected. However, this is an important trade-off in reducing false positives, firstly because HMMs detect proteins with greater sensitivity than traditional BLAST methods (38) and secondly because defence system proteins often comprise domains that are ubiquitous in other molecular systems. To aid in the identification of multi-gene systems split between contigs, we developed several relaxed system classifications (specified in [system]_other.yaml files) that require only two defence genes to be present and co-localised. The raw HMMER outputs can also be inspected, allowing users to identify potential orphan defence genes or highly divergent homologues.

Using PADLOC, we identified several clusters of Doron system genes that had strong associations with additional proteins. Based on these associations, we propose new types of Druantia, Hachiman, Lamassu, Septu, Thoeris, and Zorya systems. Septu type II was recently discovered independently and classified as a Type I-A bacterial retron (14,57,58). Members of the Type I-A retrons include Ec73 from *E. coli* and Vc95 from *Vibrio cholerae*, which provide defence against phages (14,58). Our detection of Septu type II demonstrates the capability of our approach for identification of variant defence systems. Recently, a type I Thoeris defence system, comprised of ThsA and ThsB, was shown to generate an isomer of cyclic adenosine diphosphate ribose (v-cADPR) from NAD^+^ in response to phage infection (61,62). It is proposed that v-cADPR is a second messenger that triggers further degradation of NAD^+^ by ThsB to induce cell death (62). As a putative HIT family nucleotide hydrolase/transferase, we hypothesise that ThsC of the newly identified Thoeris type II systems might play a role in the formation or degradation of the v-cADPR second messenger to regulate NAD^+^ degradation, perhaps as an off-switch analogous to the RING nucleases associated with some type III CRISPR-Cas systems that use cyclic oligonucleotide signalling (63–65). ZorA and ZorB from Zorya systems share sequence similarity with the inner membrane flagella motor proteins MotA and MotB, respectively (13). However, ZorAB are not sufficient for defence and it has been proposed that a ZorAB complex forms a proton channel that facilitates abortive infection, whereas ZorC, ZorD, and ZorE perform additional essential roles as phage sensors or activators of ZorAB (13). Since our data demonstrate activity of the Zorya type III system comprised of ZorA, ZorB, ZorF and ZorG, we propose that ZorF and ZorG function are regulators of ZorAB activity in place of ZorC, ZorD and ZorE. From the other new system types we identified, DruL, HamC, and LmuC comprise domains of unknown function. An NMR structure for the HamC protein of *Rhodospirillum rubrum* ATCC 11170 has been solved (PDB ID: 2K0M; DOI: 10.2210/pdb2K0M/pdb), with similar topology to Nuclear Transport Factor 2 (66). However, the function of HamC in phage defence remains unknown. Altogether, the data presented here extend the spectrum of potential defence systems and provide a foundation for further experimental study of their mechanisms.

The discovery of new defence systems is progressing rapidly, and importantly PADLOC can be updated to incorporate these systems as they are characterised. Using our modular approach to the organisation of HMMs and system classifications, defence systems can be easily added or updated as required. For greater accessibility, we have also developed a PADLOC webserver that allows users to analyse their genomes of choice or browse a pre-computed database of defence systems identified in RefSeq genomes. PADLOC is an open-source project, with code, HMMs, and system classifications available on GitHub. Additional curation of high quality HMMs for additional defence systems will be required to establish PADLOC as a comprehensive resource for defence system identification. We encourage the community to submit new defence system data for addition to the PADLOC database.

## DATA AVAILABILITY

The defence systems identified in this study can be viewed on the PADLOC webserver (https://padloc.otago.ac.nz). Additional genomes can be searched for defence systems by submitting them on the webserver or by downloading PADLOC from GitHub and running the software locally (https://github.com/padlocbio/padloc).

## Supplementary Material

gkab883_Supplemental_Files
