# Supplementary material for: Identification and classification of antiviral defence systems in bacteria and archaea with PADLOC reveals new system types
Source: Nucleic Acids Res. 2021 Oct 4;49(19):10868–78. doi: 10.1093/nar/gkab883 (PMC8565338; doi:10.1093/nar/gkab883)
Supplement: gkab883_Supplemental_Files [file gkab883_supplemental_files.zip › payne_2021_supplementary_revised_R2.pdf]

Identification and classification of antiviral defence systems in bacteria and archaea with PADLOC reveals new system types

Leighton J. Payne, Thomas C. Todeschini, Yi Wu, Benjamin J. Perry, Clive W. Ronson, Peter C. Fineran, Franklin L. Nobrega, Simon A. Jackson

Supplementary information

A

druantia_type_I.yaml

```
maximum_separation: 3
minimum_core: 5
minimum_total: 5
core_genes:
  - DruA1
  - DruB1
  - DruC1
  - DruD1
  - DruE1
optional_genes:
  - NA
prohibited_genes:
  - NA
```

hmm_meta.txt (excerpt)

hmm.name	protein.name	e.val.thresh...	hmm.cov...	target.cov...
DruA1_00001	DruA1	1e-05	0.3	0.3
DruA1_00002	DruA1	1e-05	0.3	0.3
DruB1_00001	DruB1	1e-05	0.3	0.3
DruB1_00002	DruB1	1e-05	0.3	0.3
DruB1_00003	DruB1	1e-05	0.3	0.3
DruC1_00001	DruC1	1e-05	0.3	0.3
DruC1_00002	DruC1	1e-05	0.3	0.3
DruD1_00001	DruD1	1e-05	0.3	0.3
DruD1_00002	DruD1	1e-05	0.3	0.3
DruD1_00003	DruD1	1e-05	0.3	0.3
DruD1_00004	DruD1	1e-05	0.3	0.3
DruD1_00005	DruD1	1e-05	0.3	0.3
DruE1_00001	DruE1	1e-05	0.3	0.3

B

hmm_meta.txt (hypothetical; excerpt)

hmm.name	protein.name	author	literature.ref	database.ref
cas_TM1811_Csm1	Cas10a	Haft DH	10/ggdjdf	TIGRFAM; TIGR02578
Type_V_V_U1	Cas12	Makarova KS	10/ggkfgj	NA
shah_acc_0001	Csx1/Csm6	Shah SA	10/ggqv9p	NA
Cas12a_M11	Cas12a	Russel J	10/gshm	NA
COG1336	Cmr4	Makarova KS	10/ggkfgj	COG; COG1336
pfam00078	RT	Eddy SR	10/ggkfgj	PFAM; PF00078
padloc_Cas13a	Cas13a	Jackson SA	NA	NA

Figure S1. PADLOC system models are structured to be simple and easy to update. (**A**) By using a central HMM metadata file, one line of a PADLOC system definition model can refer to multiple HMMs, each with individual HMM scoring and coverage thresholds (which can help to fine-tune models). (**B**) The HMM metadata file also allows for acknowledgement of authorship, links to the studies from which sequences or HMMs were obtained – via their digital object identifier (DOI), and reference to the original name of the HMM if it was obtained from an existing database. These attribution fields will be particularly useful in keeping track of HMM origin when adding additional well-studied systems to the database. For example, many HMMs have already been built for CRISPR-Cas systems by several groups and shown to be effective.

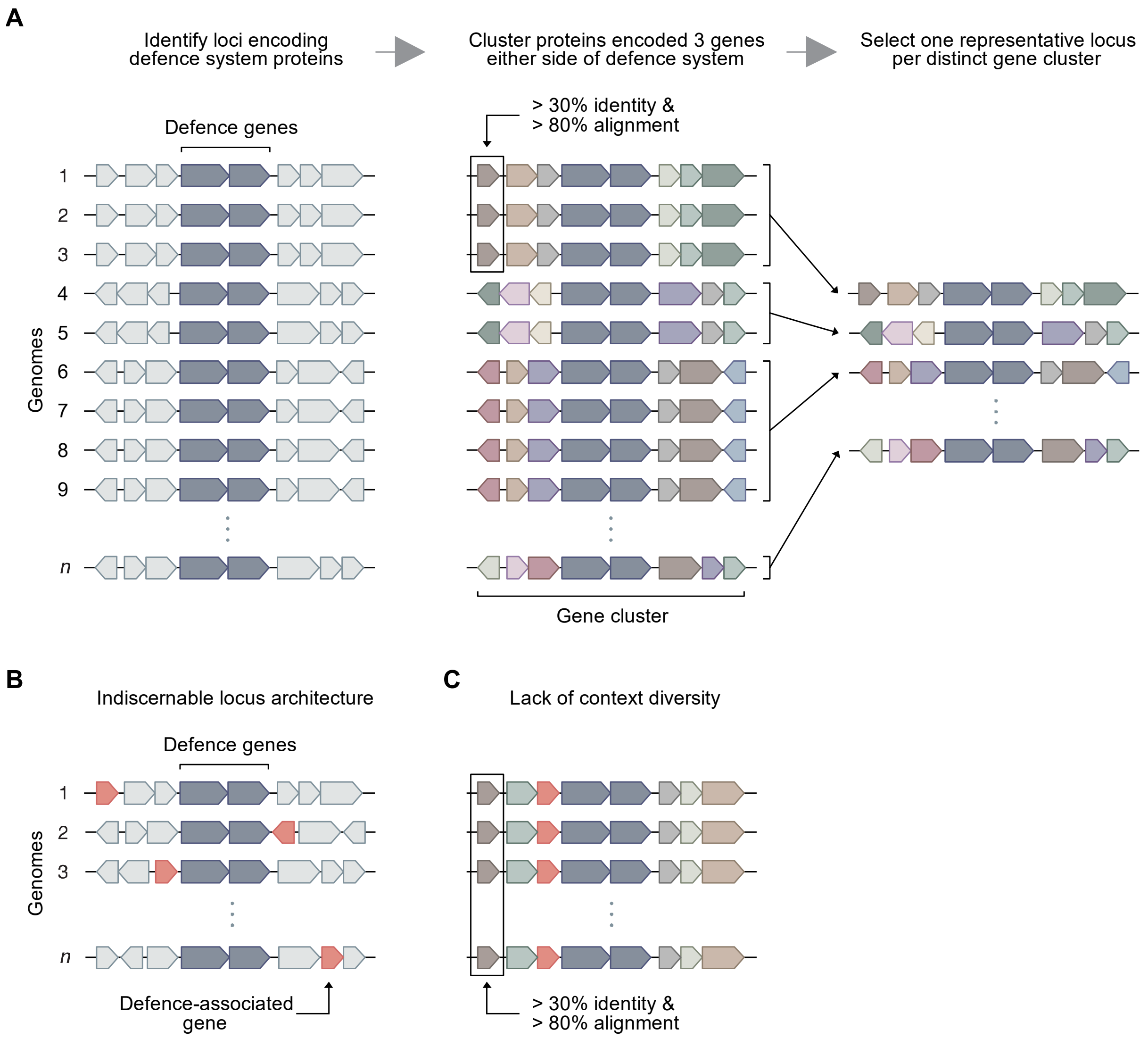

Figure S2. Selecting representative loci and subsequent filtering of putative defence systems. (**A**) One representative of each distinct gene cluster was used when calculating association frequencies between genes. This approach was taken to prevent overvaluation of frequent associations that could falsely arise from overrepresentation of common organisms in our dataset due to sequencing bias. Briefly, the proteins encoded up to three genes either side of each identified defence gene cluster identified were clustered into groups of homologues. One representative was selected per gene cluster based on this grouping. (**B**) Putative defence system variants with indiscernible locus architecture and (**C**) systems which lacked context diversity (based on nearest three genes on either side of the defence system) were excluded from further analysis. Defence system genes are shown in dark grey. Putative defence-associated proteins are shown in red.

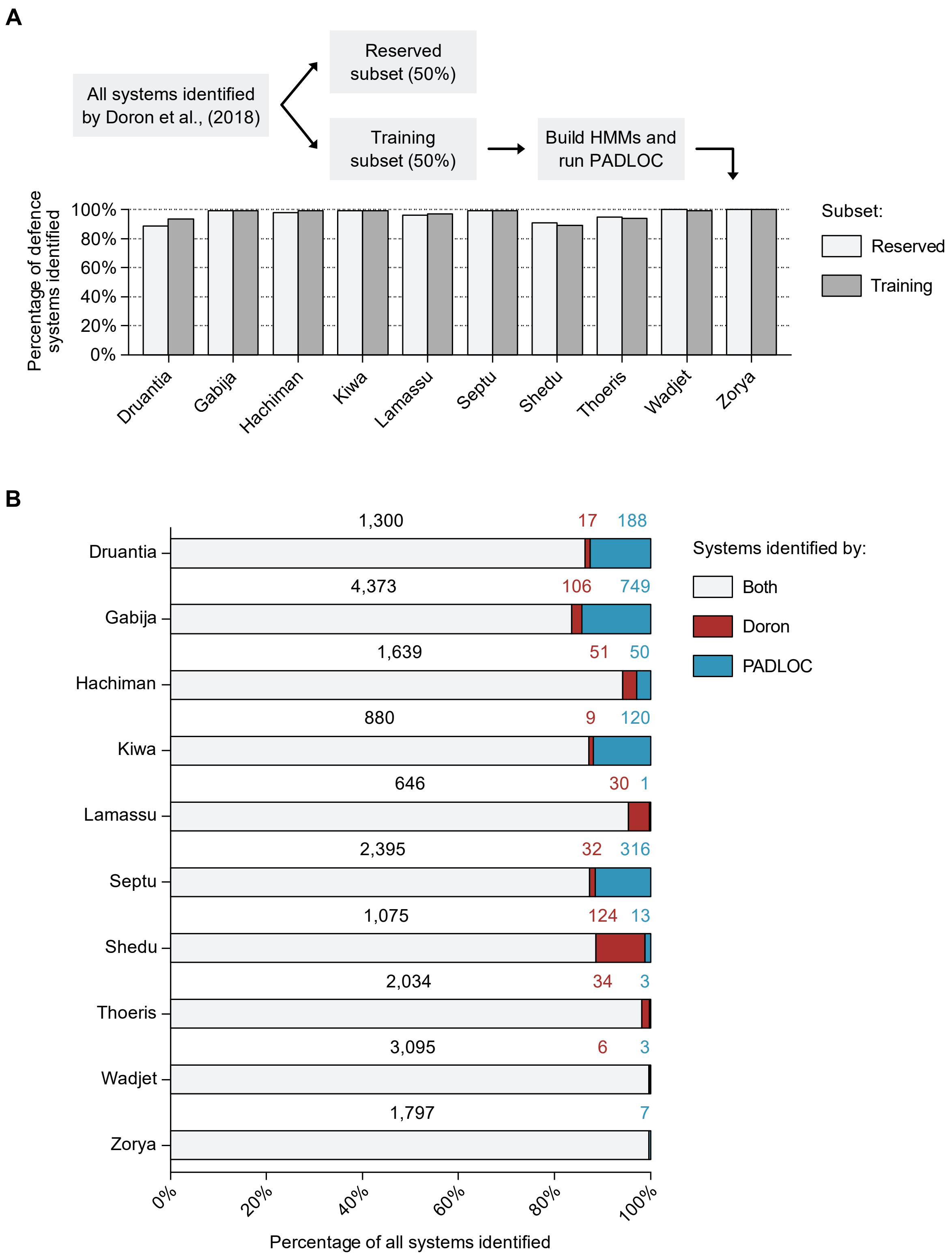

Figure S3. Defence systems are identified with high accuracy using PADLOC. (**A**) Percentage of known systems listed in Doron *et al.* that were successfully recalled by PADLOC, using only data from a random training subset (50%) for each type of system. (**B**) Percentage of all systems identified in the genomes listed in Doron *et al.* (Doron et al., 2018) and successfully recalled by PADLOC (grey), missed by PADLOC (red) or identified by PADLOC but not Doron *et al* (blue). Total system counts are show above bars.

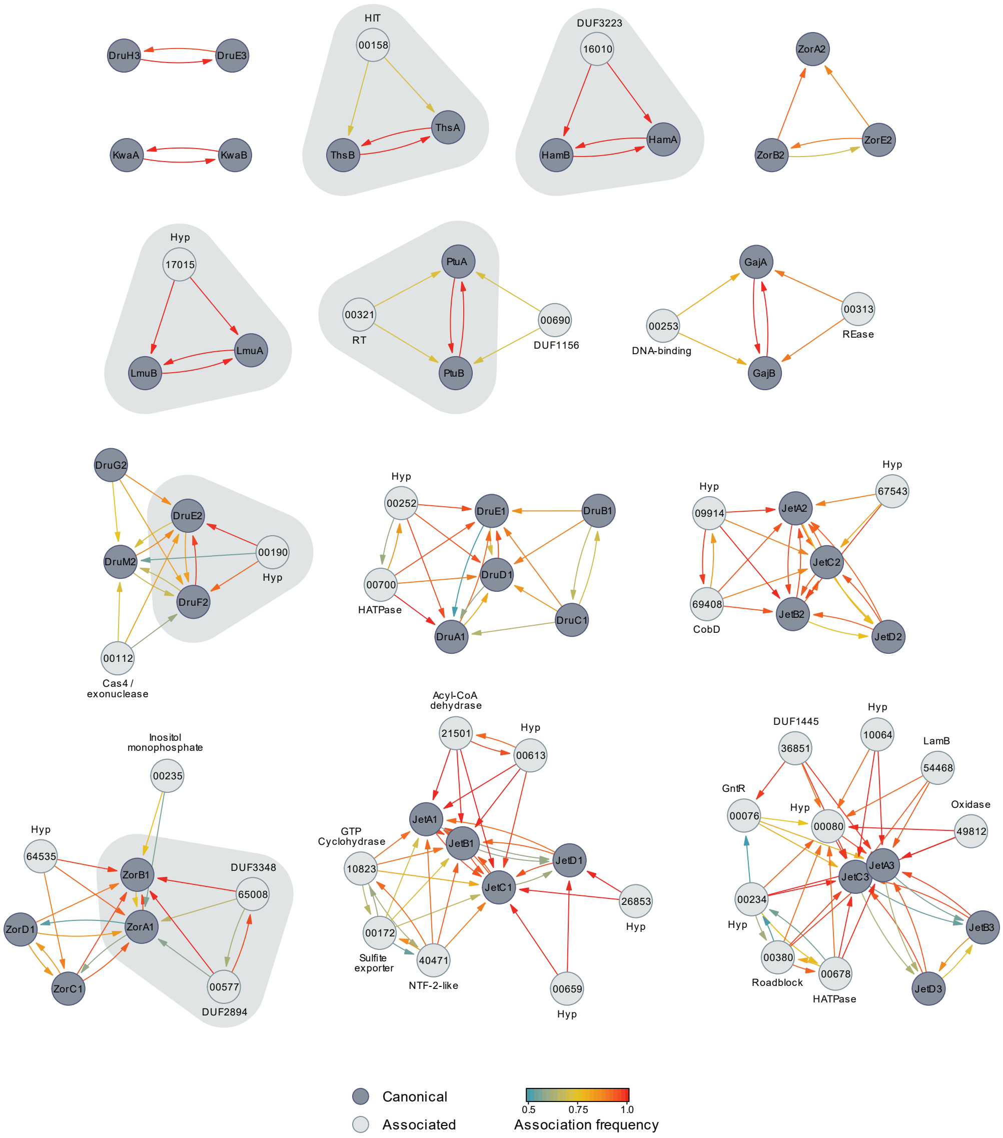

Figure S4. Additional proteins are associated with Doron system gene clusters. Associations shown are after filtering for abundance greater than 50 and association frequency greater than 0.5. Dark grey nodes are canonical Doron system proteins. Light grey nodes are Doron-associated proteins. Associations that exhibited features characteristic of defence systems (as described in the main text) are highlighted by a grey background. Domains: RT, reverse transcriptase; DUF, domain of unknown function; TIR, Toll-interleukin receptor; HIT, Histidine triad motif; REase, restriction endonuclease; Hyp, hypothetical protein.

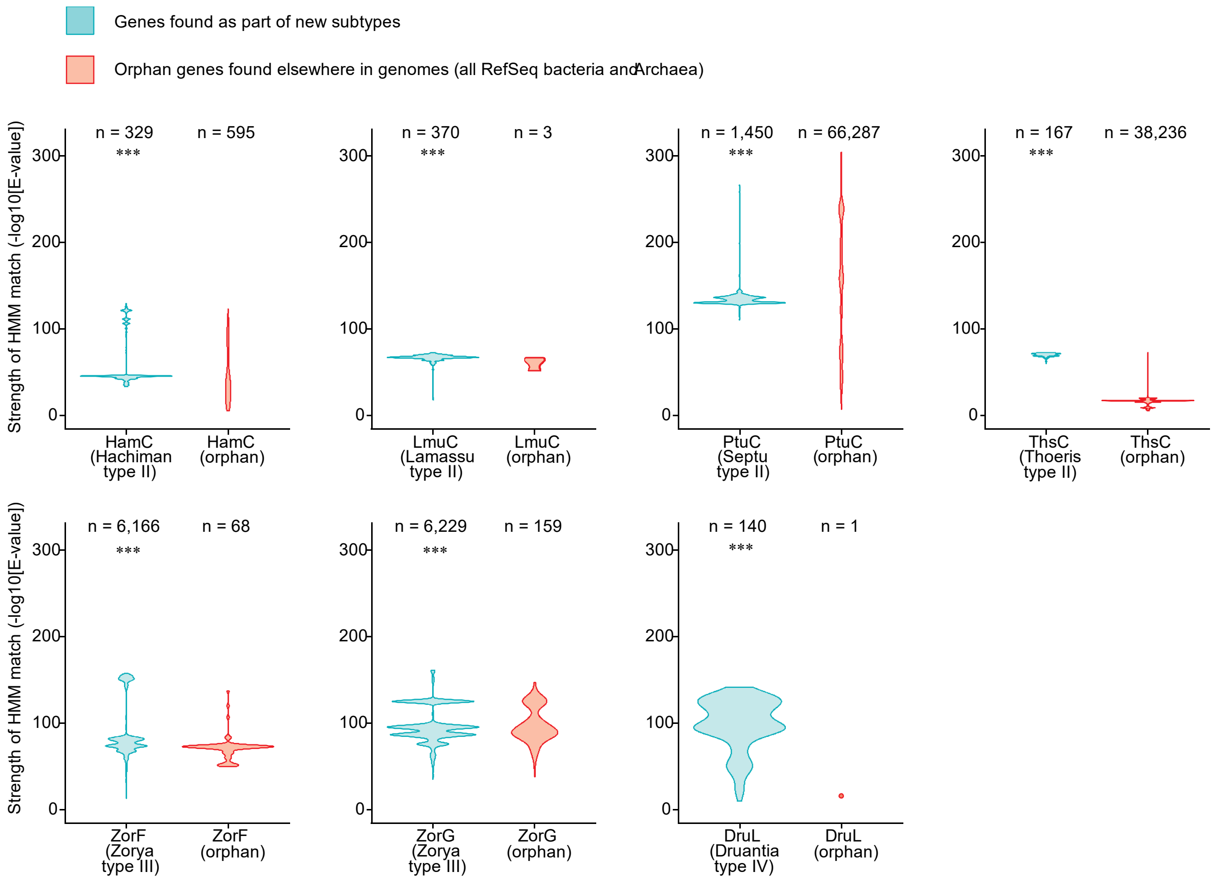

Figure S5. The lack of orphan genes encoding proteins that are closely to the Doron variant subunits demonstrates the new Doron subtype classifications are typically robust. All RefSeq v201 genomes were searched for Doron system-associated proteins. The E-values of the resulting hits are shown for Doron system-associated proteins identified as part of their respective variant system versus elsewhere in the genome (orphans). Hits were filtered for those with HMM and target alignment coverages above 0.75 to limit false positives. *n* = number of instances identified. Orphans generally were matched by their respective HMMs with a higher E-value (i.e. were weaker hits), indicating that the orphan genes were more divergent. Proteins with common domains, such as ThsC (HIT domain) and PtuC (RT domain) are found more often as orphans due to the ubiquity of these domains. Some of the orphan genes are also likely due to fragmentation of defence systems in incomplete genomes due to contig breaks. In all cases the observed associations were significant (*** $p < 0.001$), determined using one-sample proportion tests of the null hypothesis “that the observed number of associations with new subtypes is not different to the expected random probability”. The expected random probability was based on the total number of ‘base’ Doron loci observed for each system type (e.g. the number of Hachiman type I and type II systems combined), and a conservative estimate (higher being more conservative) on the total number of unique genes searched, as 100,000 relatively unique genomes (accurate de-duplication of closely related species/genome assemblies is non-trivial), encoding an average of 4,000 genes each.

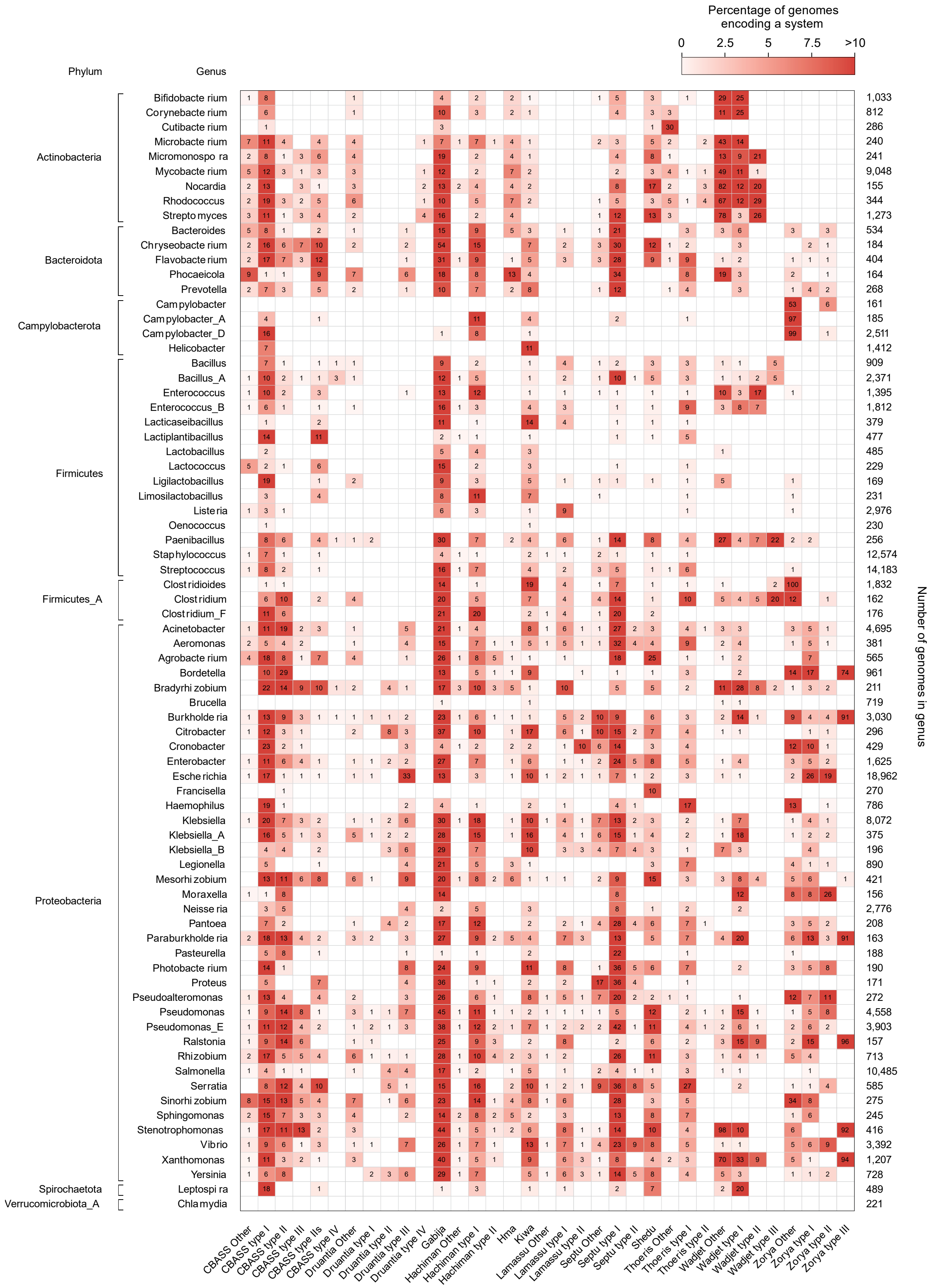

Figure S6. Abundance of Doron system variants in prokaryotic genomes identified with PADLOC. All genomes from RefSeq v201 Archaea and Bacteria were searched with PADLOC. The values in the boxes represent, for each genus, the average percentage of genomes in each species encoding a system, grouped using GTDB taxonomy (Parks et al., 2020). The colouring provides a visual

representation of these prevalence values. Shown are phyla/genera with more than 150 genomes (as such, not all phyla displayed in the main text figure are included here). See **Supplementary Table S5** for system prevalence in all genera.

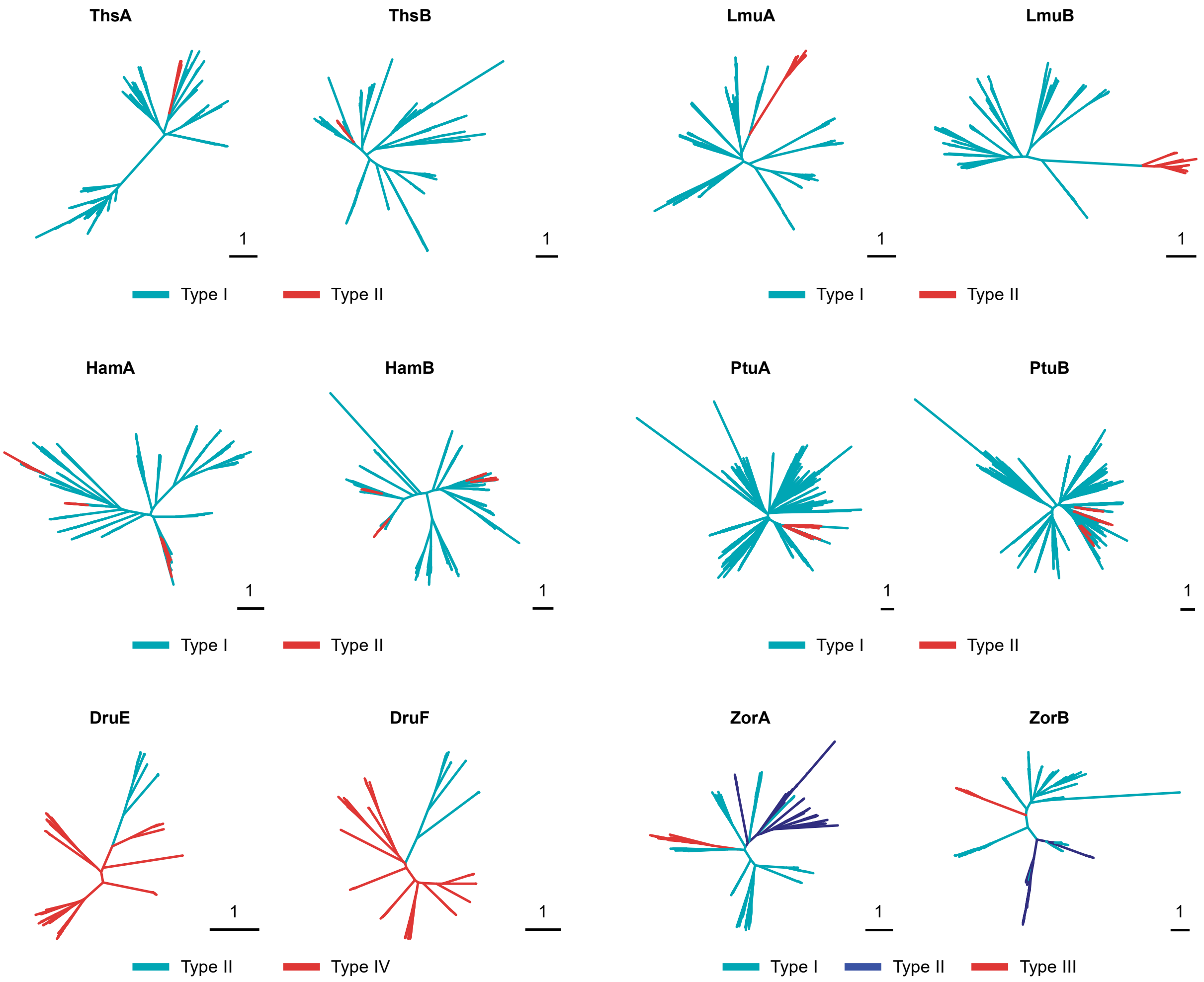

Figure S7. The proteins shared between the canonical and new defence system types are divergent. Trees were built from a sample of the sequences from the systems identified in all of RefSeq v201 (as described in methods). The sequences used to build the trees are listed in **Supplementary Table S4**. Branches are coloured based on the subtype of system to which the protein belongs. Branch length represents average amino acid substitutions per site. Trees represent the consensus of 1000 ultrafast bootstrap replicates.

Table S1. Primers used in this study.

Primer	Sequence	Description
**pDefense**		
FN0031	TTTAAAGCGGCCGCTCTACTAGCGCAGCTTAATTA	Amplification of plasmid backbone containing p15A origin of replication and Cm^R selection marker (derived from pACYC-Duet1 Novagen) for *E. coli*, adding SbfI and NotI restriction sites upstream of the multiple cloning site (MCS).
FN0032	TATATACCTGCAGGCTCCTTATTAAAGTTAAACAAAATTATTTC	
FN0126	TTTAAAGGATCCTCTACTAGCGCAGCTTAATTA	Amplification of plasmid backbone containing p15A origin of replication and Cm^R selection marker (derived from pACYC-Duet1 Novagen) for *E. coli*, adding KpnI and BamHI restriction sites upstream of the MCS.
FN0127	TATATAGGTACCAACTCCTTATTAAAGTTAAACAAAATTATTTC	
**Variant defence systems**		
FN0128	TATATAGGTACCATGGCGAAAGCTTCAACACGG	Amplification of Zorya III from DSM 12575.
FN0129	AAATTTGGATCCCTATTTTTTCCTGCGTCCGCC	
FN0035	TATATACCTGCAGGCTGTCCGGGATCACATCTGAA	Amplification of Hachiman II from DSM 14551.
FN0036	AAATTTGCGGCCGCCGATCGGCGAAAACGTCAGAC	
FN0037	TATATACCTGCAGGATGGGCACCCTCCATGACAAA	Amplification of Lamassu II from DSM 9628.
FN0038	AAATTTGCGGCCGCTCAATCGCCAATGAAATCGCC	

Table S2. Plasmids used in this study.

Vector	Origin	Resistance	Description
pUOS001	p15A	Cm^R	pDefense, derivative of pACYC-Duet1 (Novagen) with added SbfI and NotI restriction sites upstream of the MCS.
pUOS014	p15A	Cm^R	pDefense, derivative of pACYC-Duet1 (Novagen) with added KpnI and BamHI restriction sites upstream of the MCS.
pUOS002	p15A	Cm^R	pZorya, derivative of pUOS001 with inserted Zorya III from DSM 12575.
pUOS003	p15A	Cm^R	pHachiman, derivative of pUOS014, with inserted Hachiman II from DSM 14551.
pUOS004	p15A	Cm^R	pLamassu, derivative of pUOS014, with inserted Lamassu II from DSM 9628.

Table S3. Example loci for putative new defence systems

System	Organism	Assembly accession	Sequence accession	Location
Druantia type IV	*Streptomyces pratensis* ATCC 33331	GCF_000176115.2	NC_016114.1	1503581..1512208
Hachiman type II	*Vibrio parahaemolyticus* S159	GCF_000489295.1	NZ_AWHU01000046.1	6244..2203
Lamassu type II	*Escherichia coli* 401675	GCF_001266375.1	NZ_JHTG01000020.1	16393..19801
Septu type II	*Escherichia coli* DEC1A	GCF_000249055.1	NZ_AIEV01000030.1	150717..153954
Thoeris type II	*Pseudomonas aeruginosa* AZPAE15057	GCF_000793205.1	NZ_JTMP01000006.1	9236..11036
Zorya type III	*Burkholderia mallei* ATCC 23344	GCF_000011705.1	NC_006349.2	1161204..1166418
Hma	*Magnetococcus marinus* MC-1	GCF_000014865.1	NC_008576.1	3438205..3447483
