# Supplementary material for: Identification and classification of antiviral defence systems in bacteria and archaea with PADLOC reveals new system types
Source: Nucleic Acids Res. 2021 Oct 4;49(19):10868–78. doi: 10.1093/nar/gkab883 (PMC8565338; doi:10.1093/nar/gkab883)
Supplement: gkab883_Supplemental_Files [file gkab883_supplemental_files.zip › Supplementary_table_legends.docx]

**Supplementary Table S4:** Protein sequences used to build the trees in Supplementary Figure S7.

**Supplementary Table S5:** Abundance of defence systems identified with PADLOC in bacteria and archaea. All genomes from RefSeq v201 Archaea and Bacteria were searched with PADLOC. The values in the table represent, for each genus, the percentage of genomes encoding a system, grouped using GTDB taxonomy (44).
